# A prospective multi-country observational trial to compare the incidences of diabetic ketoacidosis in the month of Ramadan, the preceding month, and the following month (DKAR international)

**DOI:** 10.1186/s40200-016-0272-4

**Published:** 2016-11-05

**Authors:** Elamin I. E. Abdelgadir, Mohamed M. Hassanein, Alaaeldin M. K. Bashier, Sulaf Abdelaziz, Salwa Baki, Asma Chadli, Sara Askaoui, El Ansari Nawal, Ines S. Slim, El Mghari Ghizlane, Khadija Hafidh, Fatheya Alawadi

**Affiliations:** 1 Dubai Hospital, Dubai Health Authority, Alkhaleej Road, P.O. Box 7272, Dubai, United Arab Emirates; 2 Suba University Hospital, Khartoum, Sudan; 3 CHU Ibn Rochd- Faculty of Medicine and Pharmacy- Hassan II University, Marrakesh, Morocco; 4 Mohamed VI University Hospital, Marrakesh, Morocco; 5 Farhat Hached University Hospital, Tunisia, Tunisia; 6 Mohamed II University Hospital Casablanca, Casablanca, Morocco

**Keywords:** Ramadan, DKA, Length of stay, Length of acidosis, Shaaban

## Abstract

**Background:**

1.7 billion Muslims worldwide obey divine commands of fasting for a month. This may increase the probability of the acute complications of diabetes during the fasting period.

**Design and methods:**

We primarily aimed to compare the incidences and duration of Diabetic ketoacidosis (DKA) admissions during Ramadan compared to the month before (Shaaban) and the month after (Shawal) as well as the average pre-Ramadan six months' admissions. Our secondary objective was to assess the different incidence of DKA between Ramadan and the other months regarding precipitating factors, fasting practices in people admitted with DKA and gender differences.

This was a prospective study that included all Muslims who were admitted with DKA to major hospitals in the United Arab Emirates, Sudan, Tunisia and Morocco during the pre-Ramadan month, Ramadan and post-Ramadan month, in addition to the average monthly admissions during the last six months before Ramadan. Demographics, clinical, and laboratory indices were collected and analyzed to assess primary and secondary end points.

**Results:**

One hundred seventy patients were admitted during the study duration, 56 were admitted during Ramadan and 63 in Shawal. Six months before Ramadan showed an average admission of 56 + 7 per month. All those admitted during Ramadan were people with type1 diabetes. 29.8 % of those admitted during Ramadan did not receive structured education program on diabetes management in Ramadan. Non-compliance to medications represented the commonest cause for admission in the whole study period. Hospital stay was comparable through different months, but the duration of acidosis was longest during Ramadan month.

**Conclusion:**

In concordance with DKAR1, DKAR international showed higher rates of DKA during Ramadan when compared to preceding Lunar month (Shaaban). In Shawal, however, the rates of DKA admission were higher than the average monthly DKA admissions. The duration of acidosis was longer in Ramadan group and positively correlated with duration of diabetes. Many patients did not receive structured education about diabetes and fasting Ramadan. Our study calls for formal pre-Ramadan education and enforces the need for advice against fasting in patients who already experienced DKA in the months preceding Ramadan.

## Background

Ramadan is the ninth month of the Islamic calendar and is observed by Muslims worldwide as a month of fasting to commemorate the first revelation of the Quran to Prophet Muhammad according to Islamic belief. This month lasts 29–30 days and Muslims fast for 14–18 h a day. Fasting is obligatory for adult Muslims. Sick people, those who are traveling and those who cannot tolerate fasting are exempt from this duty.

While fasting from dawn until sunset, Muslims refrain from consuming food and drinking liquids, which puts diabetic people at high risk of acute complications, namely hyperglycemia, hypoglycemia and diabetic ketoacidosis (DKA) and increased hospital admissions as was seen in the EPIDIAR study [1].

Worldwide; 90 % of diabetes among adults is type 2 diabetes mellitus [2, 3]. It is estimated that 346 million people worldwide have diabetes and more than 80 % of diabetes deaths occur in low- and middle-income countries [4].

Diabetes has several serious complications, and DKA is one of them. Because of absence or deficiency of insulin, the body cannot use glucose for energy and thus uses fats as an alternative. Consequently, ketone bodies accumulate as a waste of this process and lead to DKA. This occurs in almost 3.3 % of type 1 diabetic patients [5, 6] and was the leading cause of death in them [7]. Furthermore, reports from Health and Social Care Information Centre showed the likelihood of mortality within 21 months after having an incidence of DKA is 2.764 fold than normal population [8]. Nonetheless, almost third of DKA patients (31 %) might have another admission within a year with DKA, and this might further increase the risk of mortality and morbidity [9].

The ADA (American Diabetes Association) consensus categorized DM (Diabetes Mellitus) patients with a history of DKA and Hyperosmolar non-kenotic state within the past 3 months or patients with poor glycemic control as high risk and advised them not to fast. Despite this, people with diabetes insist on fasting [10].

Data on DKA during Ramadan is still to be enriched by the research. Some of the authors of this paper have previously assessed the incidences of DKA in the United Arab Emirates. Results of that article showed longer hospital stay during Ramadan for DKA patients and higher rates of DKA during the last 6 months in the Ramadan group. However, the rate of DKA admissions during Ramadan was less than the following months [11].

Sudan, being one of the countries that participated in the trial, had a prevalence of DM in 2006 of 19.2 %. The 2010 annual statistics reports issued from Sudan, Ministry of Health showed 16 % of hospital admissions were due to diabetes. More than 60 % of diabetic patients attending the private sector had various complications, 10.1 % were DKA [12]. Prevalence of diabetes in the United Arab Emirates is reaching 19 % according to the International Diabetes Federation records. The prevalence of diabetes in Tunisia was reported to be 9.5 % and ranged between 12.3 in urban areas compared to 5.6 in the countryside [13]. In Morocco, the prevalence of diabetes was reported to be 6.6 % [14].

Data about admission rates during the month of Ramadan remains limited; the first paper ever on diabetes and Ramadan were written in 1979 [15]. Since then only a few papers have been released to the medical library.

## Methods

### Study design

We performed this multi-center retro-prospective observation trial at six centers distributed over four middle eastern countries. The centers involved were Dubai Hospital (UAE), Rashid Hospital (UAE), Suba Teaching Hospital (Sudan), Hassan II University hospital (Morocco), Mohamed VI University Hospital (Morocco) and Farhat Hached University Hospital (Tunisia).

All hospitals follow the international recommendations for management of diabetic ketoacidosis, though some have minimal modifications to adapt it to local experience and available resources. All data were gathered by site investigators, who verified the accuracy of the data by cross-checking with electronic database, whenever available. Data from different centers were collected in a single excel sheet for analysis.

All the authors had access to the final results and vouch for the fidelity of the trial to the protocol. The first and third authors wrote the first draft of the manuscript, which was revised and approved by all the authors, who also assume responsibility for the accuracy and completeness of its content and for the decision to submit the manuscript for publication.

### Patients

All patients admitted during the months of Ramadan of the year 2016 (1437 Hijri), were observed during their hospital stay. All patients were eligible for inclusion in the trial regardless of the type of diabetes, renal function, comorbidities, and severity of ketoacidosis.

### Procedures

During Hospital stay, DKA was managed as per protocol. Data collected included patient's demographics (age, gender, and nationality), details of diabetes (the type of diabetes, duration of disease, degree of control), precipitating factors for DKA, the length of acidosis, the length of hospital stay, and any diabetes-related comorbidity.

Patients' files were then reviewed to look for any admissions with DKA over the preceding six months. In case the patient had been admitted before, similar data to that collected during admission was collected and charted in the data collection sheet.

All patients were then followed for two months to observe for any further admissions with DKA. The Same information was collected from patients who were admitted during the month of Shawal.

Plasma glucose was measured by glucose oxidase method, glycosylated hemoglobin (HbA1C) by High-performance liquid chromatography (HPLC) method, blood Ketones by biosensor method and urinary acetone was measured by strip-based on the nitroprusside reaction.

### Outcomes

#### Primary outcome

We primarily aimed to compare the incidences and duration of DKA admissions during Ramadan to the month before (Shaaban) and the month after (Shawal). We also aimed at comparing the admission during Ramadan to the total admission with DKA in the preceding six months.

#### Secondary outcomes

Our secondary objective was to compare precipitating factors of DKA, fasting practices in people admitted with DKA and gender differences between Ramadan and the other months.

Demographics, clinical, and laboratory indices were collected and analyzed to assess primary and secondary end points.

### Definitions

We defined the duration of acidosis as the time from admission to hospital to time of resumption the subcutaneous insulin.

We diagnosed DKA according to the following criteria:

We are adopting the Joint British Societies guideline for the management of DKA [16]

- Ketonemia (3 mmol/L and over), or significant Ketonuria (more than 2+ on standard urine sticks).
- Blood glucose over 11 mmol/L or known diabetes mellitus.
- Bicarbonate below 15 mmol/L and venous PH less than 7.3.

#### Statistical analysis

All data was entered in an excel sheet, and the statistical calculation was done by IBM computer using SPSS (statistical program for social science version 12.0.). In the analysis, we ran a descriptive analysis of the data using multiple tests including Chi-square, Fisher exact, and Unpaired exact test. In our analysis P value of <0.05 was considered significant [17].

## Results

Total Number of patients who were admitted with DKA among all centers was 170 patients, 57 during Ramadan, 50 during Shaaban and 63 during Shawal, with an increment rate of 12 and 21 % from Shaaban (Before Ramadan), to Ramadan, to Shawal (after Ramadan), respectively. The mean monthly admission over the preceding six months in 2015 across the three centers for DKA was 56 + 7. We will label the different groups as a pre-Ramadan group (Shaaban), Ramadan group, and Post-Ramadan group (Shawal).

The gender distribution was almost the same through the different months; men were 46 %, 44 %, and 50.7 % respectively during the three different months with a *p*-value of 0.56. None of the pre-Ramadan group was fasting. Only 19 (33.3 %) of Ramadan and 13 (20.7 %) of Post-Ramadan patients were fasting *p* <0.05.

Seventeen patients (29.8 %) of the Ramadan group did not receive a clear instruction not to fast, as well as 31 (49.2 %) of the post-Ramadan group *p* = 0.000. Only 56 % of Ramadan and 41.3 % of the post-Ramadan group received a clear instruction not to fast or received a structured education about fasting and diabetes *p* < 0.05. The rest of the patients were not sure about the pre-Ramadan instructions.

**Fig. 1:**
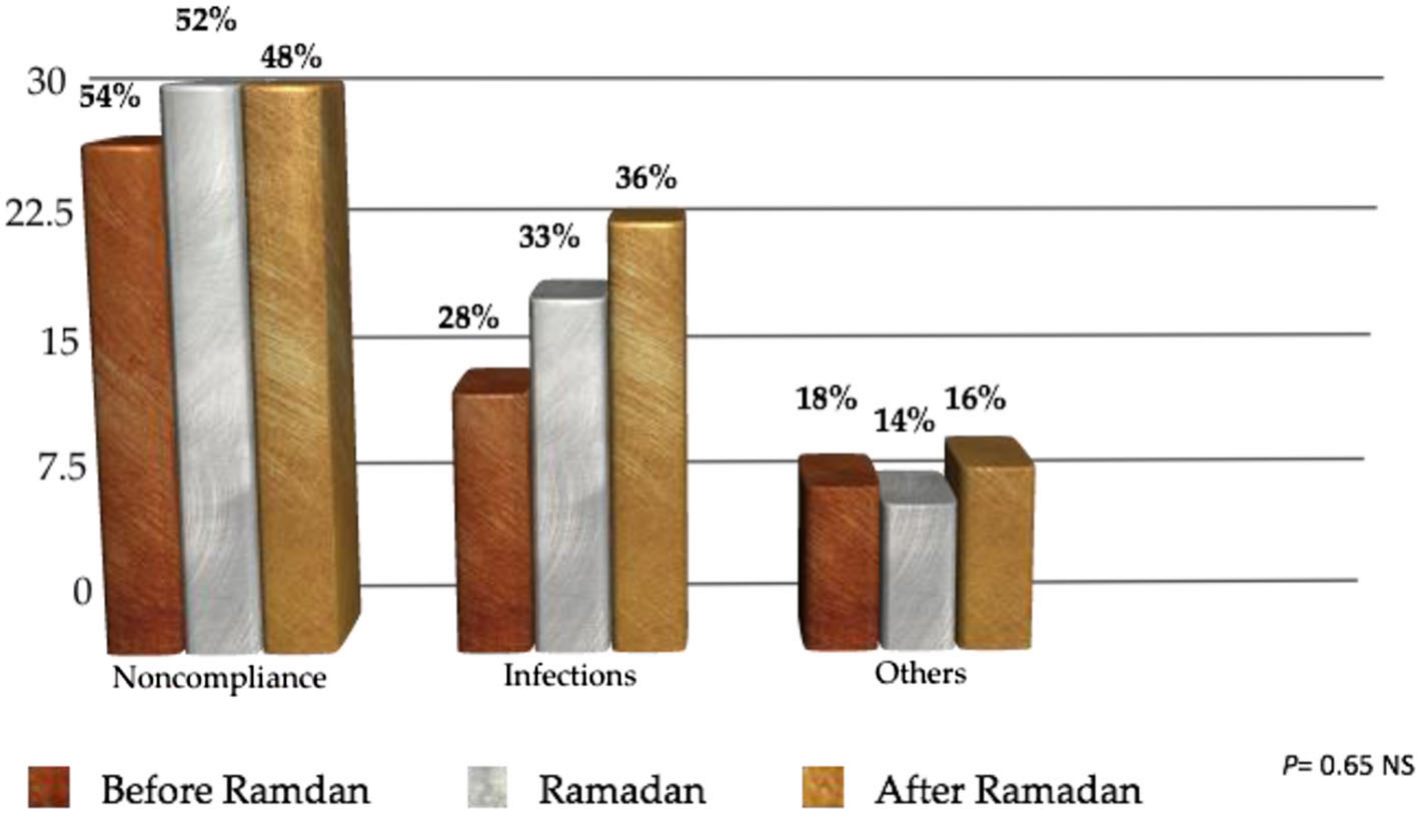
This figure shows no significant difference between the three groups

Non-compliance is the primary precipitating factor during Ramadan and other months. There is no significant difference (*p* = 0.65) (Fig. 1).

Mean HbA1C, duration of diabetes and hospital stays were not remarkably different among the groups. While the length of acidosis was highest during Ramadan 23.8 + 3 h, while it lasted only for a mean of 13.2 + 4 h in pre-Ramadan, and 23 + 6 h in post-Ramadan *p* = 0.02 (Table 1).

**Table 1:** Comparison between the studied groups regarding the duration of DM, HbA1C, hospital stay and acidosis duration​

Variables	Shaaban (before) *N* = 50	Ramadan *N* = 57	Shawal (After) *N* = 63	*X* ^2^	*P*
HBA1C	11.5 ± 3	11.4 ± 2.4	10.6 ± 2.2	1.4	0.36NS
Duration of DM (years)	6.8 ± 3	8.9 ± 3	8.9 ± 4	1.4	0.26NS
Duration of acidosis (hours)	13.2 ± 4	23.8 ± 3	23 ± 6	4.5	0.02S Bef. versus Du. (*p* = 0.01S) Bef. Versus Aft. (*p* = 0.03S) Du. Versus aft. (*p* = 0.77NS)
Hospital stay (days)	4.9 ± 2	5.1 ± 2.2	4.5 ± 1.7	1.3	0.27NS

This table showed longer duration of acidosis during Ramadan in comparison to other months. HbA1c, Duration of hospital stay, and duration of DM among different groups did not show difference​

Fifteen patients (29.1 %) of Ramadan group had a history of DKA in the previous six months, while 12 patients (24 %) of the pre-Ramadan group and 12 patients (25 %) of the post-Ramadan group had the DKA in the preceding six months *p* = 0.97.

In Ramadan group, the longer the duration of DM, the more extended the period of the acidosis *p* = 0.03, but that was not the case upon the correlation of the Age and the HbA1C (Table 2).

**Table 2:** Correlation between duration of acidosis versus different variables among group during Ramdan

Variables	Acidosis duration	
	*r*	*P*
Age	0.01	0.66
Duration of DM	0.46	0.03
HBA1C	0.10	0.54

This table shows statistically significant positive correlation between duration of acidosis and duration of DM by using correlation coefficient test. No significant correlation versus other variables

None of our DKA patients required referral to the intensive care units (ICU) after being admitted to the medical ward, and there was no mortality among our total number of patients.

## Discussion

The current study is a multicenter retro-prospective observational trial that looked at the incidence of DKA over a three months' period (a month before Ramadan, Ramadan, and a month after). Compared to our previous study (DKAR1) we have included patients admitted during the month of Shaaban to have a better representation of the admissions in the months before and after Ramadan [11]. To ensure accuracy and consistency in data collection all participating centers used same diagnostic criteria for DKA diagnosis as well as the same performa for data collection. Moreover, more centers participated in the current trial compared to the previous study that included only three centers from UAE. Seven centers representing the leading governmental institutions, and university hospitals in Dubai, Sudan, Tunisia, and Morocco have participated in this trial, giving the study the power to better reflect the actual incidence of DKA during the three months' period of the Trial.

Most patients do present with typical features of diabetic ketoacidosis during Ramadan. However, Baş VN et al. have reported a case of euglycemic DKA (blood glucose of <200 mg/dl, and ketonemia) in a 14-year-old newly diagnosed type1 diabetes who was fasting during Ramadan. Bas VN et al. case emphasizes the importance of evaluation of acid–base state, urine glucose, and ketone values in all type 1 diabetic patients during Ramadan. [18]. Another case of DKA during Ramadan was reported Friedrich I et al. who concluded that omitting doses during fasting as well as dehydration predispose patients to develop DKA. Besides, fasting accelerates development of lipolysis and ketosis and increases glucagon levels. Thus, these pathophysiological aberrations related to fasting in ketosis-prone patients endanger metabolic control in type1 diabetes [19].

Very few studies assessed the incidence of DKA during Ramadan. Al-Alwan et al. have studied 20 patients with type1 diabetes aged 8–14 years. Diabetes duration in the studied group was more than one year. Despite their age, 12 of them fasted during Ramadan. None of those patients was admitted with DKA during Ramadan [20]. Another study from Libya by Elmehdawi R. et al. retrospectively analyzed admissions through 2007 and concluded that the incidence of DKA during Ramadan is significantly less than the other lunar months (*p* = 0.001). They further reported no difference in neither the length of hospital stay nor mortality [21]. In DKAR1 we reported higher incidences of DKA during Ramadan when compared to the admissions in the preceding six months [11]. The current study did not support the same conclusion; as the mean number of admissions in the six months preceding Ramadan was 56+ 7, while during Ramadan the total number admitted was 57 patients.

In the DKAR1 study we noticed an increase in the admissions with DKA during the month of Shawal (post-Ramadan month); this observation was confirmed in the current study in which the total number of patients admitted with DKA during Shawal was 63 versus 57 in Ramadan. There is no clear explanation for this observation. However, it might reflect the worse glycemic control during Ramadan brought forward to the month of Shawal, or it might simply be a reflection for post-Ramadan festivities days, where plenty of high calories recipes are provided.

It was proved in many studies that structured education before Ramadan improves diabetes outcomes, an example of these trials is READ study and Ramadan Prospective Diabetes Study [22–24]. Moreover, structured education has further reduced incidences of hypoglycemia during Ramadan in patients with low risk of hypoglycemia [25], this could easily be attained by pre-Ramadan consultation and education since the pre-Ramadan assessment might discover the patients at risk of complications as well as training them how to avoid them [26]. In our cohort, 29.8 % of patients admitted during Ramadan stated that they did not receive any specific education or instructions on fasting.

Our findings are consistent with Elmehdawi group in that there was no significant difference in hospital stay when comparing Ramadan and other months. However, in this study the duration of acidosis was longer during Ramadan (23.8 + 3 h), while it lasted only for a mean of 13.2 + 4 h in Shaaban, and 23 + 6 h in Shawal (*p* = 0.02). Furthermore, the duration of acidosis correlated well with the duration of diabetes; the longer the duration of diabetes the longer was the period of acidosis (*p* = 0.03).

Unlike DKAR1 in which the most common cause of hospital admission was infections, in the current study, the main reason for DKA was non-compliance.

### Limitations

The study included patients from Gulf (UAE) and North Africa; we did not have patients to represent Muslims from the Indian subcontinent or Southeast Asia. However, the large sample size makes it the largest study ever that evaluated the incidence of DKA in Ramadan.

## Conclusions

In concordance with DKAR1, DKAR international showed higher rates of DKA during Ramadan when compared to preceding Lunar month (Shaaban). In Shawal, however, the rates of DKA admission were higher than the average monthly DKA admissions. The duration of acidosis was longer in Ramadan group and positively correlated with duration of diabetes. Many patients did not receive structured education about diabetes and fasting Ramadan. Our study for calls structured pre-Ramadan education and enforced the need for advice against fasting in patients who already experienced DKA in the months preceding Ramadan.

## Abbreviations

ADA: American Diabetes Association
DKA: Diabetic ketoacidosis
READ: Ramadan education and awareness in diabetes study
